# HEARTS quality: a policy framework to strengthen hypertension and cardiovascular risk management in primary healthcare—insights from HEARTS in the Americas

**DOI:** 10.1016/j.lana.2025.101311

**Published:** 2025-12-01

**Authors:** Esteban Londoño, Reena Gupta, Patrick Van der Stuyft, Martin Heine, Gloria Giraldo, Grace Marie Ku, Jeffrey Brettler, Andrés Rosende, Vilma Irazola, Jerry Toelsie, Carolina Neira, Teresa Aumala, Yamilé Valdés, Eric Zúñiga, Libardo Rodríguez, Matías Villatoro, María Cristina Escobar, Vivian Pérez, Alied Bencomo, Michael Pereira, Andelys de la Rosa, Pedro Ordunez

**Affiliations:** a Department of Noncommunicable Diseases and Mental Health, Pan American Health Organization, Washington, D.C., USA; b Resolve to Save Lives, New York, USA; c University of California at San Francisco, San Francisco, USA; d Faculty of Medicine and Health Sciences, Ghent University, Belgium; e Department of Global Public Health & Bioethics, Julius Center for Health Sciences and Primary Care, University Medical Center, Utrecht, the Netherlands; f Department of Noncommunicable Diseases and Mental Health, Pan American Health Organization, Subregional Program Coordination Caribbean, Barbados; g Department of Public Health, Institute of Tropical Medicine, Antwerp, Belgium; h Kaiser Permanante Bernard J. Tyson School of Medicine, Los Angeles, CA, USA; i Department of Research in Chronic Diseases, Institute for Clinical Effectiveness and Health Policy, Argentina; j Department of Physiology, Anton de Kom University, Suriname; k Department of Noncommunicable Diseases, Ministry of Health, Santiago, Chile; l Secretaría de Salud del Distrito Metropolitano de Quito, Ecuador; m Instituto de Endocrinología, Ministerio de Salud Pública, La Habana, Cuba; n Servicio de Salud, Antofagasta, Universidad de Antofagasta, Antofagasta, Chile; o Oficina de Enfermedades No Transmisibles, Ministerio de Salud, San Salvador, El Salvador; p Organización Panamericana de la Salud, Santiago, Chile; q Organización Panamericana de la Salud, Ciudad de México, Mexico; r Technical Officer for Treatment and Management of Cardiovascular Diseases (HEARTS). Ministry of Health, Georgetown, Guyana; s Ministerio de Salud, Santo Domingo, Dominican Republic

**Keywords:** Americas, Hypertension, Cardiovascular diseases, Primary health care, Quality improvement, Health systems strengthening, Chronic disease care

## Abstract

HEARTS in the Americas is the largest-scale implementation of the WHO's global initiative, with 33 countries participating, 28 having adopted standardized clinical pathways, and about 10,000 primary healthcare facilities engaged. Despite progress, fragmented care, limited availability of validated blood pressure devices, restricted access to essential medicines, and weak quality assurance systems continue to hinder hypertension control and cardiovascular risk management. In response, PAHO and participating countries co-developed the HEARTS Quality Framework. Grounded in regional implementation, this model synthesizes global evidence and lessons from Latin America and the Caribbean. Co-designed by Ministries of Health, care providers, and international experts, it translates HEARTS strategies into actionable system-level objectives. Clearly defined outcome indicators and implementation targets promote institutionalization, quality improvement, and primary healthcare strengthening—supporting HEARTS scale-up and equitable outcomes. With appropriate contextualization, the HEARTS Quality Framework provides a practical roadmap for countries beyond the Region to advance primary healthcare-based chronic disease care.

## Introduction

Cardiovascular diseases, primarily ischemic heart disease and stroke, are the leading cause of death in the Americas, accounting for over 2.2 million deaths annually and contributing substantially to premature mortality.^1^ Cardiovascular diseases also impose a significant economic burden by raising healthcare costs, hindering economic growth and exacerbating social inequities in the Region.^2^^,^^3^ The absolute number of cardiovascular diseases cases has continued to rise, while the decline in mortality has slowed since the mid-2010s,^4^ jeopardizing progress toward the goal of reducing premature mortality from non-communicable diseases by 30% by 2030.^5^

Although effective interventions for cardiovascular diseases are available—particularly for hypertension, its leading risk factor—most health systems have not implemented them at scale.^6^^,^^7^ Reactive and fragmented models of care persist, resulting in misdiagnosis, inadequate risk assessment, non-standardized treatment, limited health worker training, and weak monitoring systems.^8^ This underscores the urgent need to strengthen health systems through a primary health care approach,^9^^,^^10^ as the foundation for effective and equitable non-communicable diseases prevention and management.

Hypertension, which affects up to 40% of adults, is the leading population-attributable cause of cardiovascular diseases worldwide.^11^ However, major care gaps persist in the Americas, including inadequate access, limited healthcare coverage, and deficiencies in quality of care. Currently, 30% of people with hypertension remain undiagnosed, 14% of those diagnosed do not receive antihypertensive treatment, and 40% of those treated do not achieve blood pressure control. As a result, the overall population-level control rate (defined as blood pressure <140/90 mmHg) stands at just 36%.^1^

HEARTS in the Americas represents the regional adaptation of the World Health Organization (WHO) Global HEARTS initiative and its largest implementation worldwide. However, despite progress, most countries are still far from the target of 50% hypertension control at the population level.^12^ Without addressing persistent health system and operational barriers, the potential for sustained, high-quality implementation remains uncertain, as does the opportunity to save millions of lives.

A structured quality framework is essential to accelerate implementation by enhancing scaling-up and ensuring that expanded coverage translates into measurable population-level outcomes. Building on extensive experience from diverse country contexts, this paper introduces the first regional HEARTS Quality Framework—a structured model designed to guide and strengthen the implementation and institutionalization of the HEARTS quality improvement approach across the Americas.

This health policy paper begins by outlining the foundations of HEARTS in the Americas, followed by a description of its quality improvement methodology. It then introduces the HEARTS Quality Framework, which defines a shared vision and mission, along with a set of programmatic strategies to guide national implementation. The paper also presents successful cases of HEARTS implementation across diverse settings in Latin America and the Caribbean, and concludes with a forward-looking perspective on how the quality framework can help advance cardiovascular health and the management of related chronic conditions.

## Policy background

Initially launched in four countries in mid-2017, HEARTS in the Americas established a structured model aimed at improving hypertension control, aligned with WHO efforts to standardize cardiovascular diseases management in primary healthcare (PHC).^13^^,^^14^ Since then, it has progressively expanded both within countries and across the region. While grounded in the WHO HEARTS technical package,^13^ the initiative has evolved into a comprehensive PHC intervention centered on continuous quality improvement.^8^ As part of this evolution, the Pan American Health Organization (PAHO), in collaboration with the World Hypertension League, developed the foundations of a monitoring and evaluation framework for hypertension control programs.^15^ Subsequently, HEARTS in the Americas identified key drivers for hypertension control and cardiovascular risk management,^16^ and outlined how the program is designed to support health system transformation to improve population-level outcomes in PHC settings.^17^

HEARTS in the Americas is rooted in the principles of primary health care and guided by a strong commitment to health equity. It aligns with WHO policies and declarations that position primary health care as essential for achieving universal health coverage^10^^,^^18^ and emphasizes the central role of high-quality care in advancing equity.^19^, ^20^, ^21^ HEARTS is also instrumental in implementing WHO's global action plans for the prevention and control of non-communicable diseases,^22^ and contributes to Sustainable Development Goal 3.4, which aims to reduce premature mortality from these diseases.^5^ It is additionally aligned with the PAHO's initiative Better Care for Non-communicable Diseases,^23^ which seeks to strengthen PHC operations to promote the integrated and effective management of chronic conditions.

HEARTS in the Americas operationalizes the key components of the Chronic Care Model^24^^,^^25^ into practice to strengthen PHC through improved management of chronic diseases.^26^ It also draws on successful international experiences in population-level hypertension control, particularly the Canadian hypertension program^27^ and the Kaiser Permanente initiatives in California.^28^, ^29^, ^30^

Although primarily focused on hypertension control and cardiovascular risk management, HEARTS in the Americas does not operate as a vertical program or in parallel to existing health systems. Instead, consistent with the WHO health system framework of six building blocks,^31^ it takes health system strengthening as its main driver and is gradually being integrated into national health systems. Its guiding principles emphasize country ownership and sustainability by embedding HEARTS within existing integrated health service-delivery networks.^8^ The program promotes evidence-based clinical, managerial, and policy interventions to improve access, service coverage, and quality of care.^8^^,^^17^^,^^32^

## Formative process and evidence review for framework development

In March 2025, HEARTS in the Americas convened the HEARTS Quality Group to co-design a consolidated framework synthesizing experiences and lessons from program implementation and scale-up (personal communication). The group included representatives from diverse countries implementing HEARTS, with varied professional backgrounds such as primary care physicians, nurses, program managers, and clinical specialists, selected for their programmatic expertise and diverse roles across national health systems. Coordinated by PAHO's regional HEARTS team, the group brought together international experts, ministry of health officials, health system managers, frontline providers, and PAHO technical staff. Members were selected based on their technical expertise and direct involvement in national HEARTS implementation to ensure balanced representation across countries and health system levels.

## Search strategy and selection criteria

Through a series of structured in-person and virtual consultations, participants reviewed programmatic evidence and implementation practices. The key documents informing the formative review are listed in the Supplementary Box 1. These were identified primarily through recommendations from members of the HEARTS Quality Group and complemented by targeted searches in PubMed, ResearchGate, and Google Scholar using the following search terms: “HEARTS in the Americas implementation”, “Global HEARTS implementation”, “hypertension control programs”, “HEARTS initiative evidence”, “HEARTS innovations”, “blood pressure measuring”, “blood pressure measuring devices”, “cardiovascular disease management in primary care”, “hypertension treatment in primary care”, and “cardiovascular disease management in the Americas”. We limited our search to the timeframe from 2016 (when the HEARTS initiative was launched) to 2025. No language restrictions were applied. The final list of references was curated based on relevance to the scope of this health policy article. Internal reports from PAHO's Strategic Fund and national HEARTS coordination teams were also reviewed to complement the literature search.

Evidence and implementation experiences were analyzed through an iterative, consensus-driven process to identify the core components for the quality framework. Over a series of structured discussions, participants reviewed emerging themes, mapped implementation pathways, and identified operational lessons. Consensus was achieved through group deliberation and validation rounds, supported by professional interpretation to facilitate multilingual participation.

The resulting HEARTS Quality Framework is regionally grounded, operationally feasible, and designed to support national scale-up while fostering continuous quality improvement across PHC facilities. It integrates evidence, country experience, and expert consensus to ensure methodological rigor, regional applicability, and adaptability for diverse health system contexts.

## The HEARTS quality improvement methodology for PHC settings

The distinctive feature of HEARTS in the Americas is its focus on PHC, particularly on strengthening facility-level operations and building the capacity of teams to lead the transformation of hypertension and cardiovascular care within their communities. By equipping facilities and care teams with practical tools, structured frameworks, and targeted capacity building, HEARTS facilitates the integration of evidence-based interventions into routine practice and fosters a culture of continuous quality improvement. This approach acknowledges that closing gaps in cardiovascular care is fundamentally an implementation challenge,^33^ one that requires reconfiguring how services are delivered at the local level.

To support this transformation, the program promotes the full implementation of the HEARTS hypertension control drivers—a set of standardized, evidence-based recommendations drawn from successful hypertension control programs in PHC.^16^ These eight drivers, fully aligned with the 2021 WHO guideline for the pharmacological treatment of hypertension in adults,^34^ form a comprehensive operational framework implemented through a suite of improvement interventions. These interventions enable facilities to meet essential quality standards for integrated hypertension and cardiovascular risk management, with hypertension serving as both the clinical and strategic entry point, and forming the foundation for coordinating other preventive interventions.^35^ The HEARTS quality improvement methodology, described in detail elsewhere,^36^^,^^37^ provides a stepwise approach for implementing these drivers. Table 1 summarizes the core improvement interventions required at the facility level.

**Table 1:** Summary of HEARTS core improvement interventions for primary healthcare implementation.

Healthcare domain	Improvement interventions
Diagnosis	• Intensify systematic, opportunistic facility-based screening and community outreach efforts to improve hypertension detection. • Ensure accurate blood pressure measurement for timely and precise hypertension diagnosis. • Assess cardiovascular risk in all individuals with hypertension to determine blood pressure targets and identify candidates for statins and aspirin therapy.
Treatment	• Follow the standardized treatment protocol in the HEARTS Clinical Pathway to provide high-quality antihypertensive medications, preferably single–pill combinations, and additional drugs for cardiovascular risk management. • Ensure timely and appropriate treatment titration to achieve blood pressure targets (<140/90 mmHg^a^ for all persons with hypertension; <130 mmHg systolic for high-risk individuals).
Follow-up to ensure continuity of care	• Provide intensive follow-up every two to four weeks for individuals with uncontrolled hypertension, with treatment titration until blood pressure targets are achieved. • For individuals with controlled hypertension: schedule follow-up every three months for high-risk individuals and every six months for those at lower risk. • Medications are refilled for three months in all persons with controlled hypertension.
Task shifting to provide team-based care	• Blood pressure is routinely measured by trained and certified non-medical health workers. • Non-physician healthcare providers, under medical supervision, conduct follow-up visits and titrate treatment based on the HEARTS Clinical Pathway.
Performance evaluation with feedback	• Conduct monthly performance evaluations on hypertension care and control. • Provide structured feedback to the frontline healthcare team to support continuous improvement.

a mmHg: millimeters of mercury.

Implementation begins with the formation of quality improvement teams at each facility. These are small groups, typically led by the facility manager or a designated team leader, responsible for convening and training staff, engaging them in problem-solving, and fostering ownership of the change process. This participatory model, which includes community representatives, builds a shared commitment to improvement and cultivates a community of practice. Teams begin with a situation analysis and lead the implementation process using the Plan-Do-Study–Act cycle,^38^ a structured and iterative method for introducing and evaluating care delivery changes.

A central tool in this process is the HEARTS Clinical Pathway,^35^^,^^39^ the main clinical and operational instrument of the HEARTS in the Americas program. It simplifies clinical decision-making and promotes consistent, evidence-based, high-quality care. At its core is a drug- and dose-specific treatment protocol that integrates lifestyle interventions with clearly defined pharmacological steps and titration sequences.

Six elements are essential for the successful implementation of the HEARTS quality improvement methodology at the facility level. First, develop a clear implementation plan that identifies responsible personnel, available resources, and training needs. Second, systematically integrate HEARTS hypertension control drivers into clinical and managerial routine workflows. Third, document improvements using defined indicators, data sources, and a schedule for regular data entry. Fourth, hold monthly data-driven review meetings to assess progress, support teams, and guide corrective actions while evaluating their effectiveness. Fifth, provide regular feedback to staff to celebrate achievements and sustain motivation. Finally, establish communication channels to update community and institutional stakeholders and promote the visibility of results.^36^

## Integration and sustainability of HEARTS implementation

To ensure sustained integration of the program in the PHC settings, iterative quality improvement cycles of up to six months should be used. Each cycle includes three core components, informed by implementation science principles.^40^^,^^41^

First, periodic evaluation of implementation progress, guided by the HEARTS monitoring and evaluation framework, enables PHC teams to systematically assess fidelity (adherence to recommended interventions), feasibility (integration into service delivery), acceptability (perceived value by the care team), and effectiveness (measurable improvements in coverage and control).^37^

Second, a structured training and retraining plan is required to support the use of HEARTS clinical and managerial tools. This plan should reinforce core competencies through continuous education, practice-based learning, and peer-to-peer exchange.

Third, a subnational technical assistance mechanism is needed to ensure fidelity of implementation. In this model, local health authorities and service coordinators conduct quarterly, data-driven review meetings and provide technical and administrative support to PHC facilities. This includes coaching and mentorship on the Plan-Do-Study–Act process to determine whether changes are producing improved outcomes. Support is delivered through on-site or virtual visits and is reinforced by continuous communication and follow-up.

In addition, a structured cross-learning process should be established among PHC quality improvement teams. Local health authorities and sub-national program supervisors play a central role in disseminating successful changes from individual facilities to the broader program.

**Fig. 1:**
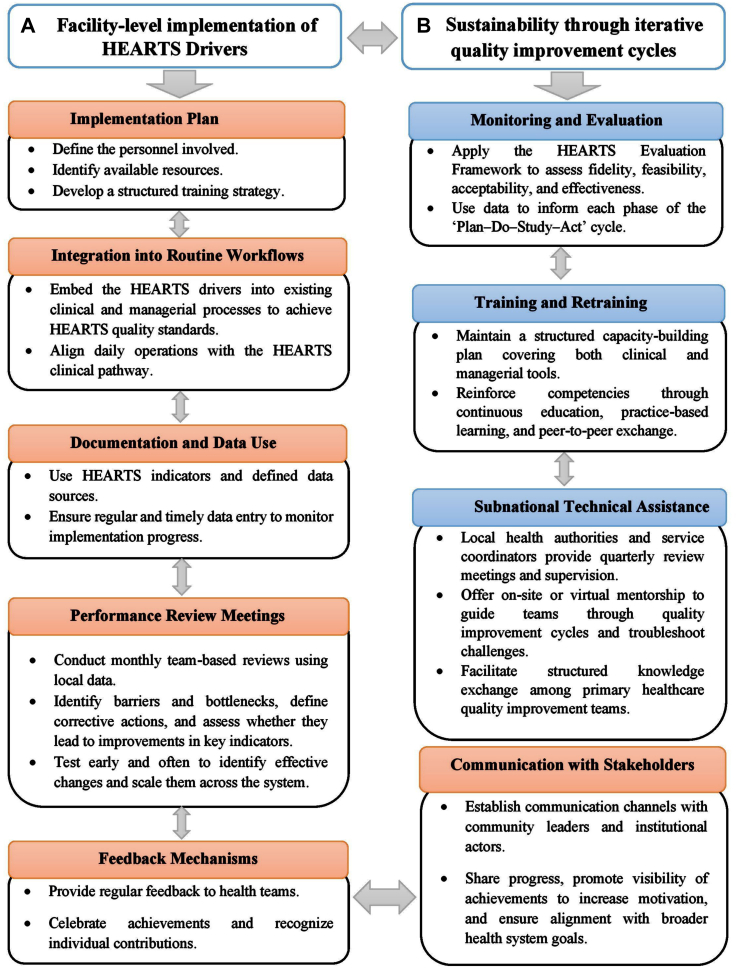
Structured process to implement and sustain the HEARTS quality interventions in primary health care settings.

Source: PAHO. HEARTS Quality Group. This figure has been developed by the authors.

Fig. 1 illustrates the overall process for implementing and sustaining HEARTS quality interventions in the PHC setting.

### The HEARTS quality framework

Quality of care is defined as "the degree to which health services for individuals and populations increase the likelihood of desired health outcomes and are consistent with current professional knowledge".^42^ It is characterized by six core attributes: effectiveness, safety, timeliness, efficiency, equity, and patient-centeredness.^43^ In the context of PHC, quality also involves first contact accessibility, coordination, comprehensiveness, and continuity,^44^ with the latter being particularly relevant for managing chronic conditions.^45^ These attributes underpin the HEARTS approach. From a health systems perspective, access, coverage, continuity, and overall quality of care are essential to improve population health outcomes. Table 2 describes how the six core attributes of quality of care are operationalized through the implementation of HEARTS in the Americas.

**Table 2:** Mapping the six core attributes of quality of care to the HEARTS in the Americas Quality Framework.

Quality attribute	HEARTS Quality Framework
Effectiveness	• HEARTS interventions are grounded in the best available scientific evidence. • Validated automated blood pressure measuring devices improve diagnostic accuracy. • Standardized treatment protocols using highly effective medications and clear thresholds for therapy initiation and intensification are aligned with World Health Organization guidance. • High cardiovascular-risk individuals receive timely preventive care. • Clinical follow-up is strengthened through management based on comprehensive cardiovascular risk assessment and defined blood pressure targets. • Digital information systems enable data-driven performance monitoring and continuous improvement in health outcomes.
Safety	• Accurate diagnosis and standardized treatment protocols ensure consistent and safe clinical management. • Clear titration procedures define minimum and maximum dosages for each medication. • Health worker training reinforces safe prescribing practices and appropriate patient monitoring. • Antihypertensive medications included in the HEARTS protocols are evidence-based, safe, and well tolerated, minimizing adverse effects. • The HEARTS Clinical Pathway establishes referral mechanisms to higher levels of care for complex cases and for individuals not controlled on full-dose triple therapy.
Timeliness	• Strengthens hypertension and cardiovascular risk detection and diagnosis within primary healthcare services to address population-level gaps in case finding. • Mandates prompt initiation of pharmacological treatment once hypertension or high cardiovascular risk is diagnosed. • Addresses therapeutic inertia by defining rapid treatment intensification when blood pressure remains uncontrolled. • Optimizes patient flow to reduce waiting times and improve access to timely, high-quality care.
Efficiency	• Standardizes treatment with a limited number of highly effective medications. • Reduces clinical variability through standardized protocols, lowering institutional costs. • Improves blood pressure control with fewer drugs and less frequent visits, reducing out-of-pocket expenses for patients and families. • Focuses on three core protocol medications and pooled procurement to lower prices and streamline supply chain management. • Promotes team-based care involving non-physician providers to enhance detection, improve control, and reduce program costs. • Uses digital information systems to increase the speed and scale of service delivery.
Equity	• Ensures access to the highest standard of cardiovascular care for all individuals, regardless of socioeconomic status or personal background. • Expands access to accurate diagnosis, essential high-quality medicines, and comprehensive hypertension and cardiovascular risk management across the Americas. • Strengthens primary healthcare as the foundation for equitable service delivery and progress toward universal health coverage.
Patient-centeredness	• Ensures that every person receives high-quality cardiovascular care within primary healthcare, tailored to individual needs and risks. • Incorporates non-pharmacological interventions adapted to each person's condition. • Promotes simplified and effective treatments with rational intervals for medication refills, reducing the clinical and logistical burden on patients and families. • Implements strategies such as multi-month refills, decentralizing care closer to people's homes, and providing treatment free of charge to reduce out-of-pocket expenses.

HEARTS in the Americas aims to serve as the institutionalized model for hypertension and cardiovascular risk management across PHC networks. The program is expanding its scope to integrate and improve the quality of cardio-kidney-metabolic care within PHC systems.^46^ Led by ministries of health, the program's mission is to strengthen PHC operations by advancing managerial and clinical practices that expand access, increase service coverage, and elevate care quality—ultimately reducing the burden of cardiovascular, renal, and metabolic diseases in the region.

HEARTS aims to achieve the 80-80-80 target^12^ by 2030: 80% of individuals with hypertension diagnosed, 80% of those diagnosed treated, and 80% of those treated achieving blood pressure control. When combined (0.80 × 0.80 × 0.80), this translates to approximately 50% overall population-level control of hypertension. Reaching this target is projected to significantly reduce cardiovascular mortality. Recent regional estimates suggest that each 1% increase in population-level hypertension control is associated with a 2.9% reduction in ischemic heart disease mortality and a 2.4% reduction in stroke mortality, both per 100,000 population in the Americas.^1^

Despite the Region of the Americas exhibiting the highest hypertension control rates among all WHO regions, progress has been uneven and remains insufficient to achieve population-level targets. In 2019, an estimated 70% of adults aged 30–79 years with hypertension were aware of their condition, 86% of those diagnosed were receiving treatment, and only 60% of those treated had their blood pressure effectively controlled.^1^ To reach the 80-80-80 target and achieve a population control rate above 50%, the Region must increase hypertension detection coverage by about 10% (from 70% to 80%) and improve control among those treated by 20% (from 60% to 80%).

**Fig. 2:**
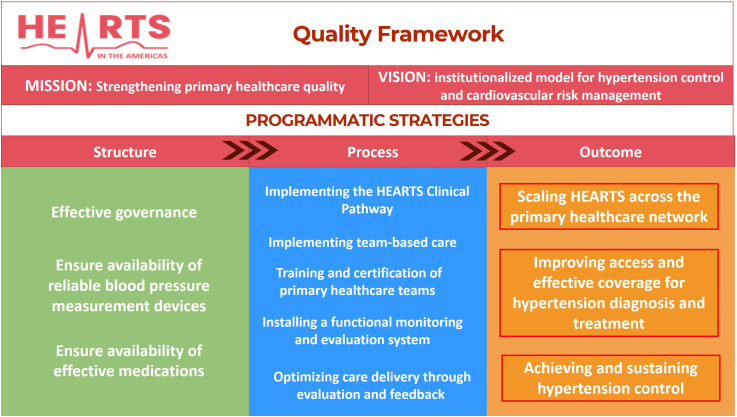
The HEART Quality Framework.

Source: PAHO. HEARTS Quality Group. This figure has been developed by the authors.

The HEARTS Quality Framework articulates the program's vision, mission, and core programmatic strategies, offering a structured approach to implementation that is aligned with national health priorities. It reinforces the connection between high-quality implementation and improved health outcomes by supporting countries in meeting intervention targets and achieving measurable results. Fig. 2 summarizes the HEARTS Quality Framework.

The programmatic strategies of the HEARTS Quality Framework are grouped into three domains: structural (foundational elements for national implementation), process (organization and delivery of services in PHC), and outcome (strategies for broader system-level impact). The following section briefly describes each strategy.

### Structure domain

#### Effective governance

Effective governance, supported by strategic leadership and a sense of urgency, is essential for implementing and scaling HEARTS in the Americas. Regional experience shows that meaningful and sustained progress depends on the coordinated and mutually reinforcing action of these actors. National steering committees guide policy development, oversee implementation, and maintain monitoring and evaluation systems. Subnational authorities (e.g., provincial, departmental, municipal) play a key role in translating national strategies into concrete actions and expanding the program across PHC services.

Moreover, assessing governance effectiveness remains challenging. The HEARTS Quality Framework promotes accountability and institutionalization, but there is a risk of complacency—especially when self-assessments obscure implementation gaps. Since external evaluations may not always be feasible or acceptable, robust, transparent, and data-driven national monitoring systems are essential for tracking governance performance. Engaging communities in governance processes and sharing quality improvement results at the facility level further strengthen transparency and accountability.

Institutionalization of the HEARTS program also depends on regulatory and operational mechanisms. These include national scale-up plans, mandatory use of validated automated blood pressure measurement devices, access to quality cardiovascular medicines, integration of and incentives to implement the HEARTS Clinical Pathway in all facilities, and task-sharing models that authorize trained non-physician health workers to adjust treatment and provide follow-up care.

#### Ensure availability of reliable blood pressure measurement devices

Blood pressure measurement is critical for diagnosing and managing hypertension, yet only about 20% of blood pressure devices globally are independently validated for accuracy.^47^^,^^48^ To ensure accurate readings, countries must adopt policies that mandate the exclusive use of clinically validated automated blood pressure measurement devices, certified by independent, non-commercial entities.^49^^,^^50^

HEARTS in the Americas supports this transition by promoting a regulatory pathway for validated blood pressure measurement devices,^51^ providing technical assistance to strengthen national regulatory frameworks, guiding procurement processes, and fostering market conditions that favor accuracy-certified devices.^51^^,^^52^ Countries that have implemented strong regulations show that clear policy and market roadmaps can accelerate progress.^53^ Still, mandates alone are insufficient. Dedicated funding and sustained investment are essential to ensure that all PHC facilities are adequately equipped with validated devices.

#### Ensure availability of effective medications

Access to quality-assured cardiovascular medicines is essential for effective hypertension and risk management in PHC settings. However, availability remains limited due to high prices, fragmented procurement, and weak supply chains. For example, in low- and middle-income countries, only 16% of eligible patients receive statins for secondary prevention of cardiovascular diseases, and just 7% for primary prevention.^54^^,^^55^ To close this gap, including single–pill combination antihypertensive therapies and high-intensity statins in national essential medicines lists for PHC can significantly enhance access and quality of care.

To reduce costs, simplify supply chains, and improve adherence, the HEARTS in the Americas pharmacologic protocols recommend rapid blood pressure control through the use of two antihypertensive medications from complementary classes, preferably as a single-pill, fixed-dose combination.^56^ To address potential limitations, the pharmacologic strategy emphasizes a limited set of long-acting core drugs produced in formulations that allow dose titration (starting with half and full doses of two medicines and/or scored tablets), as well as related single–pill combinations.

The PAHO Strategic Fund provides a pooled procurement mechanism to support access to these priority medicines and is already facilitating implementation in several countries. Through a pooled regional procurement mechanism, the PAHO Strategic Fund has defined a core set of highly effective medicines, consolidates demand across countries, negotiates long-term supply agreements with the pharmaceutical industry, and secures competitive prices for quality-assured generic products. Comparative analyses have shown that prices obtained through the PAHO Strategic Fund are 20%–99% lower than those available through national procurement systems, offering substantial opportunities for savings.^57^

Expanding access also requires updating national formularies, removing cost barriers, improving supply chain performance, ensuring market availability of recommended medicines, and strengthening procurement and distribution systems.^58^

### Process domain

#### Implementing the HEARTS clinical pathway

HEARTS promotes the use of care pathways as practical implementation tools. These pathways translate guidelines recommendations into standardized, contextualized, multidisciplinary plans that structure hypertension management, facilitate task sharing, and actively engage patients in their care.^59^

The HEARTS Clinical Pathway is the cornerstone of standardized, evidence-based care for hypertension and cardiovascular risk management. Institutionalizing its use requires not only a defined care pathway but also its consistent application in PHC facilities. This includes prescribing the recommended medications and dosages, intensifying treatment when needed, and ensuring timely follow-up.

The pharmacologic treatment protocol within the pathway is tailored by each country to permit the management of most patients with hypertension at the primary care level, while allowing referral to higher levels of care for complex cases or individuals not controlled on full-dose triple therapy. This adaptive design promotes country ownership and the delivery of consistent, evidence-based care while maintaining clinical judgment and patient-centered flexibility at both the country and facility levels.

Effective implementation depends on key enablers such as trained health personnel, validated blood pressure measurement devices, and uninterrupted access to essential medications. Establishing accountability mechanisms and incentives that promote team engagement in continuous quality improvement supports sustained application. These elements ensure the operational capacity to deliver pathway-based care consistently and at scale.^60^

Adherence to the pathway should be monitored through clinical audits that assess whether treatment decisions and follow-up frequency align with protocol recommendations.

#### Implementing team-based care

Supported by a strong body of evidence,^61^, ^62^, ^63^ HEARTS in the Americas and its implementing countries have co-created a policy framework to expand team-based care^64^ and integrate pharmacists^65^ and nurses into the management of hypertension and cardiovascular risk in PHC settings, promoting their authorization and capacity to perform treatment intensification in accordance with the national HEARTS Clinical Pathway. The program also promotes the training and certification of auxiliary health workers to measure blood pressure and assist in screening efforts.^64^

A comprehensive team-based model also recognizes patients and caregivers as active participants in care. Empowering individuals with the knowledge, skills, and enabling conditions needed for self-management improves adherence and facilitates sustained lifestyle changes.^66^ Evidence shows that self-care interventions—such as home blood pressure monitoring and family involvement—contribute to improved hypertension control, underscoring the importance of integrating person-centered self-management strategies into hypertension care.^64^

#### Training and certification of PHC teams

HEARTS supports capacity-building through standardized technical resources, including self-paced online courses hosted on PAHO's Virtual Campus for Public Health (Supplementary Box 2). These courses, which address both clinical and managerial aspects of implementation, have been key to disseminating HEARTS core components. Over 1.5 million learners have enrolled to date, supported by national incentives such as continuing medical education credits system.

While online training alone cannot meet all capacity needs, it provides a scalable foundation for broad knowledge dissemination. To ensure long-term sustainability, countries are encouraged to invite universities to integrate HEARTS content into undergraduate and postgraduate programs for health professionals. This approach aims to develop a well-prepared, multidisciplinary PHC workforce^67^ capable of delivering high-quality hypertension and cardiovascular risk management.

#### Installing a functional monitoring and evaluation system

A functional monitoring and evaluation system is foundational to HEARTS, guided by the principle “no data, no program”. Managed by PHC teams at the facility level, it ensures data are relevant for timely local decision-making and continuous quality improvement. HEARTS uses standardized indicators aligned with the HEARTS evaluation framework^37^ to promote data-driven action in service delivery.^68^ To support this, the HEARTS information system based on the District Health Information Software-2 digital platform enables systematic data entry, timely reporting, and real-time dashboards. The platform is interoperable with national health information systems, collects only aggregate data without compromising confidentiality, and guarantees full national ownership and control in line with legal frameworks.

The HEARTS platform serves not only as a technical solution but also as a tool to uncover structural and managerial barriers and support adaptive responses. Political will, data quality, digital infrastructure, and transitioning from paper-based systems are essential enablers. As countries scale HEARTS, this system^68^ will be central to achieving equitable implementation and measurable improvements in cardiovascular and other non-communicable diseases outcomes.

#### Optimizing care delivery through evaluation and feedback

The HEARTS evaluation framework^37^ supports implementation monitoring through a dual and complementary approach: internal self-evaluation by PHC teams and external assessments led by local health authorities and PHC network coordinators. This structure fosters continuous learning, helps identify performance gaps, and promotes evidence-based improvement. To ensure feasibility and strengthen technical capacity, health authorities are encouraged to collaborate with academic institutions in conducting external evaluations and research.

Facilities implementing HEARTS are expected to hold regular quality improvement meetings to review data, identify challenges, and define corrective actions. Evaluation findings should feed into structured feedback loops that drive iterative improvements, address equity and quality gaps, and help overcome implementation barriers. As implementation matures, facilities should demonstrate measurable progress in service delivery and outcomes.

### Outcome domain

#### Scaling HEARTS across the PHC network

Scaling HEARTS implementation across the PHC network is essential to achieving sustainable, population-level impact. A core feature of HEARTS in the Americas is its focus on PHC facilities, given their proximity to communities, and their ability to deliver person-centered, continuous, integrated services. Nationwide scale-up allows countries to expand equitable access, strengthen continuity of care, and embed cardiovascular risk management as a routine component of comprehensive PHC. To support this process, countries can use WHO's guidance on scaling up health service innovations,^69^ which emphasizes integration into national policies and programs to ensure sustainability. The publication *HEARTS in the Americas*: *Guide and Essentials for Implementation*^70^ also provides practical tools and lessons learned to guide both vertical and horizontal scale-up at the national level.

#### Improving access and effective coverage for hypertension diagnosis and treatment

In the Americas, nearly one in three people with hypertension remains undiagnosed.^1^ Closing this gap requires systematic and proactive screening in PHC settings to improve early detection and reduce cardiovascular risk.

Evidence shows that opportunistic screening, by measuring blood pressure in all individuals attending a health facility, is the most effective approach to increase detection.^71^^,^^72^ For populations with limited contact with the health system, community outreach strategies are essential to identify undiagnosed cases.^32^

PHC facilities should demonstrate progress in identifying individuals with hypertension within their catchment areas, using local population estimates and disease burden to guide action. Ensuring that diagnosis results in sustained care requires universal health coverage policies that guarantee access, financial protection, and continuity of high-quality hypertension management.

#### Achieving and sustaining hypertension control

Hypertension control is more than just a clinical goal; it results from a comprehensive public health strategy rooted in PHC. HEARTS in the Americas is a strategic effort focused on transforming service delivery, not a short-term project or clinical trial. Achieving sustained hypertension control requires systemic changes that strengthen health system foundations, expand access and coverage, and improve the quality and continuity of care. Focusing only on hypertension control rates without ensuring the quality of services and equity in outcomes—as reflected in structural and process strategies and indicators—can undermine long-term impact and may ultimately create a perverse incentive that favors short-term metrics over sustainable, equitable care.

HEARTS addresses key gaps across the continuum of care, from diagnosis to long-term management. Implementing its programmatic strategies through the HEARTS Quality Framework is essential to institutionalize high-quality services, increase control rates, and reduce the overall cardiovascular burden.

#### Demonstrating impact and driving health system transformation

HEARTS is delivering measurable results. Following its initial implementation in a single polyclinic in Barbados in 2014, the program expanded to Chile, Colombia, and Cuba in 2017. Currently, 33 countries in the Americas had joined the initiative, 28 had institutionalized a standardized clinical pathway, and 12 had scaled implementation to more than 80% of their PHC networks. Today, HEARTS operates in about 10,000 PHC facilities, with more than 6 million individuals on treatment—62% of whom have achieved blood pressure control.^73^ These outcomes confirm that HEARTS is not only feasible but effective. The initiative is improving hypertension care and driving broader PHC transformation across the region (Panel 1).

Panel 1 **HEARTS in action: driving results and system change**

• **Chile**: A 12-month cohort study showed that the standardized HEARTS protocol significantly improved blood pressure control (65% vs. 37% and 41%) and treatment adherence (71% vs. 18% and 23%; p < 0.001).^74^ A complementary 10-year outcomes-based Markov model found the approach highly cost-effective, with benefits exceeding costs by year two, and a cost per Disability-Adjusted Life Year averted ($2171) well below the per capita Gross Domestic Product threshold.^75^
• **Cuba**: In Matanzas, a HEARTS demonstration project led to rapid improvements in hypertension care. Within one year (2016–2017), treatment coverage reached 94% and blood pressure control 68%, with a population-level control rate of 58%—up from a 36% national baseline in 2010.^76^
• **Colombia**: At Santa Mónica Hospital in Risaralda, a quasi-experimental study found that HEARTS implementation increased the proportion of patients with controlled blood pressure from 76.6% to 84.1% in one year. Patients adhering to the protocol were 2.7 times more likely to achieve blood pressure targets (OR 2.688; 95% CI: 1.081–6.684).^77^
• **Trinidad and Tobago**: Between 2019 and mid-2021, HEARTS expanded from 5 to 46 primary healthcare facilities (covering 45% of the public system), reaching about 550,000 people. In pilot sites, control improved across all regions, with two centers reporting gains of 16% and 21%. Hypertension coverage rose from 20% to 45% in North Central and from 17% to 29% in Tobago.^78^
• **Mexico**: In Chiapas and Sonora, HEARTS was implemented in 29 primary healthcare centers and evaluated across four semesters (2020–2021). Despite disruptions from the COVID-19 pandemic and low baseline coverage (4.3% in 2020 vs. 3.7% in 2021), 55% of centers (n = 16) improved blood pressure control by the second half of 2021.^79^ On the other hand, implementation of the HEARTS model in the states of Chiapas and Yucatán, centered on standardized protocols and task shifting, proved feasible and cost-efficient. In Chiapas, standardized protocols reduced annual medication costs by approximately 9.7%, while in Yucatán, implementation was slightly more expensive due to more intensive treatment regimens but remained overall affordable.^80^
• **Venezuela**: In La Marroquina, a rural community, implementation among 52 hypertensive individuals over four months increased blood pressure control from 11.5% to 52%. Treatment coverage reached 92%, combination therapy use rose to 72%, and the maturity index reached level 4 of 5, showing strong early progress in a low-resource setting.^81^
• **Guatemala**: A six-month pilot in 11 primary healthcare centers demonstrated high feasibility and acceptability. Medication availability increased from 60% to 81%; hypertension treatment rose significantly (+22.3 individuals/month; p < 0.001), with 50.6% blood pressure control among documented cases. Most care was delivered by non-physician health workers, despite challenges in follow-up (36%) and data capture.^82^

## Forward view: using the HEARTS quality framework to advance hypertension control and cardiovascular risk management in the Americas Region and beyond

As highlighted elsewhere,^83^ the implementation of the Global HEARTS Initiative, including HEARTS in the Americas, has shown that progress depends on five key enablers: strong government commitment to hypertension control, accessible primary healthcare and competent staff, affordable medicines and services, decentralized community-based care, and reliable, patient-centered information systems. The authors also emphasized that achieving national scale requires formal and sustained quality improvement efforts, investment in team-based care, supportive policies for healthcare decentralization, and comprehensive health worker training. These findings may also be of relevance to hypertension control strategies being developed outside the region of the Americas.

Despite important progress, persistent barriers continue to hinder effective hypertension control and cardiovascular risk management globally. Fragmented models of care, limited access to validated blood pressure measurement devices, essential medicines, and interoperable health information systems, as well as the lack of policies to support team-based care collectively undermine progress—leading to underdiagnosis, suboptimal treatment, and poor population-level hypertension control rates. Furthermore, implementation of the HEARTS initiative remains uneven both across and within countries, with PHC facilities at different stages of readiness.

The HEARTS Quality Framework offers a practical and adaptable model to support national authorities in their efforts to implement and scale HEARTS, achieve their commitments and goals, and overcome key challenges by strengthening PHC performance. It translates programmatic strategies into strategic objectives and actionable system-level reforms, supported by standardized outcome indicators and implementation targets (Table 3). Its robust quality improvement methodology fosters institutionalization, adaptive learning, and real-time performance monitoring, allowing for contextual adaptation while maintaining alignment with regional goals. As countries gain experience, the framework evolves, remaining responsive, evidence-based, and consistent with updated standards of care.

**Table 3:** HEARTS programmatic strategies: Objectives, outcome indicators, and implementation targets.

	Programmatic strategy (short name)	Strategic objective	Outcome indicator	Implementation target
1	Effective governance	Strengthen national leadership and governance to scale HEARTS implementation across the entire PHC^a^ network.	1. A national HEARTS coordination committee is established, with an implementation and scale-up plan developed and updated annually. 2. A national regulatory policy and operational plan are in place to progressively ensure the exclusive commercialization and clinical use of clinically validated automated blood pressure measurement devices. 3. Policies and mechanisms are in place to ensure the uninterrupted supply of effective, high-quality essential cardiovascular disease medicines. 4. The HEARTS Clinical Pathway is institutionally adopted as the national standard of care. 5. Regulatory or legal provisions are in place to authorize adequately trained and certified non-physician PHC workers to titrate pharmacologic treatment and conduct follow-up based on the HEARTS Clinical Pathway.	1. By [Year], a fully functional national HEARTS coordination committee is established, with an approved implementation and scale-up plan reviewed and updated at least annually. The committee oversees the national implementation of HEARTS. 2. By [Year 1], a national regulatory policy and operational plan are adopted and operationalized to ensure at least 50% exclusive procurement and clinical use of clinically validated automated blood pressure measurement devices in health facilities, increasing to 100% by [Year 3]. 3. By [Year], national policies and supply chain systems ensure the continuous availability and affordability of quality-assured, effective cardiovascular medicines across all PHC facilities nationwide, in accordance with the institutionalized HEARTS Clinical Pathway. 4. By [Year], the HEARTS Clinical Pathway is formally adopted as the national standard of care and fully integrated into routine practice across all PHC settings. 5. By [Year], regulatory and legal frameworks authorize and enable at least 80% of PHC facilities to implement task-sharing models where adequately trained and certified non-physician workers manage pharmacologic treatment titration and follow-up according to the HEARTS Clinical Pathway.
2	Validated automated blood pressure measurement devices	Ensure exclusive use of clinically validated automated blood pressure measurement devices in PHC settings.	1. Percentage of PHC facilities equipped with clinically validated automated blood pressure measurement devices. 2. Percentage of blood pressure devices in use that are automated and clinically validated.	1. 100% of HEARTS PHC facilities are equipped with automated and validated blood pressure measurement devices. 2. ≥80% of blood pressure measurement devices in use are automated and clinically validated.
3	Effective medications	Guarantee equitable and continuous access to single–pill combination antihypertensives and high-intensity statins in PHC to support the full implementation of the HEARTS Clinical Pathway approved by the country.	1. Inclusion of at least one single–pill combination antihypertensive and one high-intensity statin in the national essential medicines list. 2. Percentage of PHC facilities reporting uninterrupted availability of antihypertensive medicines. 3. Percentage of PHC facilities reporting uninterrupted availability of statins.	1. At least one single–pill combination antihypertensive and one high-intensity statin included in the national essential medicines list. 2. At least 90% of HEARTS PHC facilities report uninterrupted availability of antihypertensive medicines. 3. At least 90% of HEARTS PHC facilities report uninterrupted availability of statins.
4	HEARTS Clinical Pathway	Ensure consistent application of the HEARTS Clinical Pathway in PHC.	1. Percentage of PHC facilities that adhere to the national HEARTS Clinical Pathway by prescribing protocol-recommended medications and dosages for hypertension and cardiovascular risk management. 2. Proportion of audited patient records from PHC facilities that demonstrate appropriate treatment intensification and adequate follow-up frequency in accordance with the HEARTS Clinical Pathway.	1. At least 90% of HEARTS PHC facilities adhere to the HEARTS Clinical Pathway. 2. At least 90% of audited clinical records demonstrate proper treatment intensification and follow-up.
5	Team-based care	Expand and institutionalize team-based care for hypertension management in PHC.	1. Percentage of PHC facilities where nurses or pharmacists are authorized and enabled to titrate pharmacologic treatment based on the HEARTS Clinical Pathway. 2. Proportion of PHC facilities where trained and certified auxiliary health workers conduct standardized screening activities to identify potential cases of hypertension and cardiovascular risk.	1. 100% of HEARTS PHC facilities engage nurses or pharmacists in titration. 2. ≥80% of HEARTS PHC facilities conduct screening via trained auxiliary workers.
6	PHC team training	Institutionalize HEARTS training and certification across PHC and academic programs using HEARTS content delivered through PAHO's Virtual Campus for Public Health.	1. Proportion of PHC facilities where all staff involved in hypertension and cardiovascular risk management have completed certification in the virtual courses ‘'Automatic and Accurate Blood Pressure Measurement’, and ‘Quality Improvement for PHC Teams’. 2. Proportion of universities at the national level offering programs in medicine, nursing, pharmacy and public health that have incorporated HEARTS and/or similar courses into their formal curricula.	1. At least 80% of HEARTS PHC facilities have their staff certified in blood pressure measurement and quality improvement. 2. At least 70% of key universities or health training institutions include HEARTS-related content in their curricula.
7	Monitoring and evaluation system	Ensure that PHC services routinely collect, report, and use standardized implementation data aligned with the HEARTS program variables and indicators to guide quality improvement decisions.	Percentage of PHC facilities that regularly report standardized implementation data, either directly through the HEARTS platform or via interoperability with the national health information system.	At least 80% of PHC facilities implementing HEARTS regularly report standardized implementation data.
8	Evaluation & feedback	Systematically evaluate implementation progress and apply structured feedback mechanisms to improve care quality, close performance gaps, and progressively achieve higher levels of maturity across the HEARTS hypertension control drivers.	1. Percentage of PHC facilities that conduct structured self-evaluations of implementation quality at least every six months and define corrective actions based on the evaluation findings. 2. Percentage of PHC facilities that conduct at least two data-driven review meetings in the past semester. 3. National external evaluation of HEARTS implementation quality conducted annually, either through a nationally representative sample or in at least 10% of PHC facilities.	1. At least 80% of PHC facilities implementing HEARTS conduct structured self-evaluations of implementation quality every six months and define corrective actions based on the findings. 2. At least 80% of PHC facilities implementing HEARTS, with support from local health authorities, conduct quarterly review meetings to analyze implementation data, identify performance gaps, and define corrective actions. 3. The Ministry of Health or a designated authority conducts an external evaluation of HEARTS implementation quality at the national level at least once per year.
9	Program scale-up	Institutionalize HEARTS as the standard of care for hypertension and cardiovascular risk management in all PHC facilities, in alignment with the national scale-up plan.	Percentage of PHC facilities nationwide effectively implementing the HEARTS program among the total number of facilities targeted in the national scale-up plan.	At least 80% of PHC facilities targeted in the national scale-up plan are effectively implementing the HEARTS program.
10	Hypertension coverage	Ensure that PHC facilities implementing HEARTS achieve high coverage of diagnosis and pharmacological treatment for adults with hypertension within their defined catchment areas.	1. Percentage of adults with hypertension included in updated clinical hypertension registries within the past 12 months, among the estimated adult population with hypertension in the catchment areas of PHC facilities. 2. Percentage of adults with hypertension receiving pharmacological treatment in accordance with the HEARTS Clinical Pathway, among those included in the updated clinical hypertension registries of PHC facilities.	1. At least 80% of the estimated adult population with hypertension in the catchment areas of PHC facilities implementing HEARTS are diagnosed and recorded in updated clinical hypertension registries. 2. At least 80% of adults with hypertension included in the updated clinical hypertension registries of PHC facilities implementing HEARTS are receiving pharmacological treatment in accordance with the HEARTS Clinical Pathway.
11	Hypertension control	Ensure that PHC centers implementing HEARTS achieve and sustain blood pressure control rates among adults receiving treatment for hypertension.	1. Percentage of adults with hypertension under treatment at PHC facilities who achieve blood pressure control, defined as the most recent reading of less than 140/90 mmHg. 2. Percentage of adults at high cardiovascular risk with hypertension under treatment at PHC facilities who achieve blood pressure control, defined as the most recent systolic blood pressure reading of less than 130 mmHg.	1. At least 80% of adults with hypertension under treatment at PHC facilities implementing HEARTS have their most recent blood pressure reading of less than 140/90 mmHg. 2. At least 80% of adults at high cardiovascular risk with hypertension under treatment at PHC facilities implementing HEARTS have their most recent systolic blood pressure reading of less than 130 mmHg.

a PHC: primary healthcare.

Equity is central to the framework's design. By embedding high-quality standards and promoting standardized clinical practices, HEARTS helps ensure that all populations—especially those historically underserved—benefit equally from improvements in care. In this context, quality improvement is not only a technical priority but a public health and ethical imperative.

Implementing the HEARTS Quality Framework should be understood as a dynamic and progressive process at both national and facility levels. The quality standards provide a shared benchmark for care delivery, while the implementation strategies serve as practical drivers of improvement. However, the sustainability and scalability of HEARTS depend on key enabling conditions: adequate system capacity, political commitment, long-term financing, regulatory support, and active engagement of frontline providers and communities. Operationally, the establishment of a functional national HEARTS coordination committee, overseeing an ambitious implementation and scale-up plan that engages the primary healthcare system nationwide–rather than relying on pilot or demonstrative projects—is indispensable. Without these foundations, implementation risks fragmentation and diminished impact. Aligning HEARTS deployment with broader health-system-strengthening efforts is therefore essential.

HEARTS in the Americas presents a timely and powerful vehicle to reduce the burden of cardiovascular, kidney, and metabolic conditions through strengthened PHC. Scaling up its implementation can accelerate hypertension control, reduce premature mortality, and build a sustainable foundation for integrated chronic disease management. The HEARTS Quality Framework equips countries with a tested roadmap to institutionalize these gains. Prioritizing its adoption and scale-up is critical to achieving equitable, effective, and lasting cardiovascular health globally and across the Region.

## Contributors

EL and PO led the conceptualization and methodology, conducted the formal analysis, prepared the original draft, and provided supervision of the overall writing process. RG, PVdS, MH, GG, GK, JB, AR, VI, and JT contributed to the conceptualization and methodology, and contributed to writing – review & editing, providing critical revisions that substantially improved the original draft. CN, TA, YV, EZ, LR, MV, ME, VP, AB, MP, and AdlR contributed to the validation of the methodology and results, and to writing – review & editing.

## Declaration of interests

EL, GG, AR, LR, VP, AB and PO are staff members of the Pan American Health Organization. The authors alone are responsible for the views expressed in this publication, and they do not necessarily represent the decisions or policies of the Pan American Health Organization. The remaining authors have no conflicts of interest to declare.
