# Supplementary material for: HEARTS quality: a policy framework to strengthen hypertension and cardiovascular risk management in primary healthcare—insights from HEARTS in the Americas
Source: Lancet Reg Health Am. 2025 Dec 1;53:101311. doi: 10.1016/j.lana.2025.101311 (PMC12719693; doi:10.1016/j.lana.2025.101311)
Supplement: Supplementary Material 1 [file mmc1.pdf]

HEARTS Quality: A policy framework to strengthen hypertension and cardiovascular risk management in primary health care—Insights from HEARTS in the Americas.

Supplementary Material

Table of content

Supplementary Box 1. Selected publications on programmatic evidence and implementation of the HEARTS initiative…………………………………………………………………………………………………..………… 2

Supplementary Box 2. HEARTS virtual courses………………………………………………………………..7

Supplementary Box 2. Selected publications on programmatic evidence and implementation of the HEARTS initiative

PAHO/WHO HEARTS in the Americas: Programmatic innovations and tools for implementation

- Rosende A, Romero C, DiPette DJ, Brettler J, Van der Stuyft P, Satheesh G, et al. Candidate Interventions for Integrating Hypertension and Cardiovascular-Kidney-Metabolic Care in Primary Health Settings: HEARTS 2.0 Phase 1. Glob Heart. 2025;20(1):45.
- Irazola V, Prado C, Rosende A, Flood D, Tsuyuki R, Neira Ojeda C, et al. Expanding team-based care for hypertension and cardiovascular risk management with HEARTS in the Americas. Rev Panam Salud Publica. 2025;49:1.
- Ridley E, DiPette DJ, Gysel S, Rosende A, Campbell NRC, Ojeda CN, et al. HEARTS Pharmacy: A framework for integrating pharmacists in hypertension and cardiovascular disease risk management in primary care. Rev Panam Salud Publica. 2025;49:e35.
- Pan American Health Organization. HEARTS in the Americas: Evaluation framework for continuous quality improvement in primary care centers. Washington D.C.; 2025. https://iris.paho.org/handle/10665.2/64210.
- Ordunez P, Campbell NRC, DiPette DJ, Jaffe MG, Rosende A, Martinez R, et al. HEARTS in the Americas: Targeting Health System Change to Improve Population Hypertension Control. Curr Hypertens Rep. 2024;26(4):141–56.
- Pan American Health Organization. HEARTS in the Americas. Quality Improvement for Primary Health Care Centers. Washington D.C.; 2024. https://iris.paho.org/handle/10665.2/59308
- Rosende A, DiPette DJ, Martinez R, Brettler JW, Rodriguez G, Zuniga E, et al. HEARTS in the Americas clinical pathway. Strengthening the decision support system to improve hypertension and cardiovascular disease risk management in primary care settings. Front Cardiovasc Med. 2023;10:1102482.
- Pan American Health Organization. HEARTS in the Americas. Compendium of essential clinical tools 2023. Washington, D.C.; 2024. https://iris.paho.org/handle/10665.2/59164
- Khan T, Moran AE, Perel P, Whelton PK, Brainin M, Feigin V, et al. The HEARTS partner forum-supporting implementation of HEARTS to treat and control hypertension. Front Public Health. 2023;11:1146441.
- Ordunez P, Campbell NRC, Giraldo Arcila GP, Angell SY, Lombardi C, Brettler JW, et al. HEARTS in the Americas: innovations for improving hypertension and cardiovascular disease risk management in primary care. Rev Panam Salud Publica. 2022;46:e96–e96.
- Brettler JW, Arcila GPG, Aumala T, Best A, Campbell NR, Cyr S, et al. Drivers and scorecards to improve hypertension control in primary care practice: Recommendations from the HEARTS in the Americas Innovation Group. Lancet Reg Heal - Am. 2022;9:100223.
- Jaffe MG, DiPette DJ, Campbell NRC, Angell SY, Ordunez P. Developing population-based hypertension control programs. Rev Panam Salud Publica/Pan Am J Public Heal. 2022;46.
- Ordunez P, Tajer C, Gaziano T, Rodríguez YA, Rosende A, Jaffe MG. La aplicación HEARTS: una herramienta clínica para el manejo del riesgo cardiovascular y la hipertensión en la atención primaria de salud [The HEARTS app: a clinical tool for cardiovascular risk and hypertension management in primary health care]. Rev Panam Salud Publica. 2022;46:e46.
- Rosende A, DiPette D, Brettler J, Rodríguez G, Zuniga E, Connell K, et al. HEARTS in the Americas appraisal checklist and clinical pathway for comprehensive hypertension management in primary care. Rev Panam Salud Publica. Spec Issue HEARTS 2022;46.
- Prado P, Gamarra A, Rodriguez L, Brettler J, Farrell M, Girola ME, et al. Monitoring and evaluation platform for HEARTS in the Americas: improving population-based hypertension control programs in primary health care. Rev Panam Salud Publica. Spec Issue Hear. 2022;46.
- Cohn J, Bygrave H, Roberts T, Khan T, Ojji D, Ordunez P. Addressing Failures in Achieving Hypertension Control in Low- and Middle-Income Settings through Simplified Treatment Algorithms. Glob Heart. 2022;17(1):28.
- Pan American Health Organization. HEARTS in the Americas: Guide and Essentials for Implementation. Washington D.C.; 2022. https://iris.paho.org/handle/10665.2/55804
- Giraldo GP, Joseph KT, Angell SY, Campbell NRC, Connell K, DiPette DJ, et al. Mapping stages, barriers and facilitators to the implementation of HEARTS in the Americas initiative in 12 countries: A qualitative study. J Clin Hypertens (Greenwich). 2021;23(4):755-765.
- Campbell NRC, Ordunez P, Giraldo G, Rodriguez Morales YA, Lombardi C, Khan T, et al. WHO HEARTS: A Global Program to Reduce Cardiovascular Disease Burden: Experience Implementing in the Americas and Opportunities in Canada. Can J Cardiol. 2021;37(5):744-755.
- Skeete J, Connell K, Ordunez P, DiPette DJ. Approaches to the Management of Hypertension in Resource-Limited Settings: Strategies to Overcome the Hypertension Crisis in the Post-COVID Era. Integr Blood Press Control. 2020;13:125-133.
- Campbell NRC, Schutte AE, Varghese CV, Ordunez P, Zhang XH, Khan T, Sharman JE, et al. São Paulo call to action for the prevention and control of high blood pressure: 2020. J Clin Hypertens (Greenwich). 2019 Dec;21(12):1744-1752. Erratum in: J Clin Hypertens (Greenwich). 2020;22(2):309.
- World Health Organization. WHO package of essential noncommunicable (PEN) disease interventions for primary health care. Geneva, Switzerland; 2020. https://www.who.int/publications/i/item/who-package-of-essential-noncommunicable-(pen)-disease-interventions-for-primary-health-care
- Avezum Á, Perel P, Oliveira GBF, Lopez-Jaramillo P, Restrepo G, Loustalot F, et al. Challenges and Opportunities to Scale Up Cardiovascular Disease Secondary Prevention in Latin America and the Caribbean. Glob Heart. 2018;13(2):83-91.
- Patel P, Ordunez P, Connell K, Lackland D, DiPette D; Standardized Hypertension Treatment and Prevention Network. Standardized Hypertension Management to Reduce Cardiovascular Disease Morbidity and Mortality Worldwide. South Med J. 2018;111(3):133-136.
- Campbell NRC, Ordunez P, DiPette DJ, Giraldo GP, Angell SY, Jaffe MG, et al. Monitoring and evaluation framework

for hypertension programs. A collaboration between the Pan American Health Organization and World Hypertension League. J Clin Hypertens. 2018;20(6):984–90.
- Salicrup LA, Ordunez P, Engelgau MM. Hypertension control activities in Latin America and the Caribbean: opportunities for late-stage (T4) translation research. Rev Panam Salud Publica. 2018;42:e22.
- Campbell N, Ordunez P, Jaffe MG, Orias M, DiPette DJ, Patel P, et al. Implementing standardized performance indicators to improve hypertension control at both the population and healthcare organization levels. J Clin Hypertens (Greenwich). 2017 May;19(5):456-461. doi: 10.1111/jch.12980. Epub 2017 Feb 13. PMID: 28191704; PMCID: PMC5476944.
- Colgrove P, Connell KL, Lackland DT, Ordunez P, DiPette DJ. Controlling hypertension and reducing its associated morbidity and mortality in the Caribbean: implications of race and ethnicity. J Clin Hypertens (Greenwich). 2017 Oct;19(10):1010-1014.
- World Health Organization. Hearts: technical package for cardiovascular disease management in primary health care. 2016. https://www.who.int/publications/i/item/9789241511377.

Blood pressure measuring devices

- Hernández Véliz D, Valdés González Y, Armas Rojas NB, De la Noval García R, Ringrose J, Padwal R. Validation of combiomed hipermax-BF model A7101 automatic oscillometric upper-arm sphygmomanometer in general population: AAMI/ESH/ISO universal standard (ISO 81060-2:2018/Amd 1:2020). J Hum Hypertens. 2024 Nov;38(11):779-785.
- Desson Z, Sharman JE, Searles A, Schutte AE, Delles C, Olsen MH, et al. Improving the accuracy of blood pressure measuring devices in Australia: a modelled return on investment study. J Hum Hypertens. 2024 Feb;38(2):177-186.
- Lombardi C, Picone DS, Sharman JE, Campbell NRC, Farias R, Guerre S, et al. Country experiences on the path to exclusive use of validated automated blood pressure measuring devices within the HEARTS in the Americas Initiative. J Hum Hypertens. 2023;37(2):120–5.
- Whelton PK, Picone DS, Padwal R, Campbell NRC, Drawz P, Rakotz MK, et al. Global proliferation and clinical consequences of non-validated automated BP devices. J Hum Hypertens. 2023;37(2):115–9.
- Ordunez P, Lombardi C, Picone DS, Brady TM, Campbell NRC, Moran AE, et al. HEARTS in the Americas: a global example of using clinically validated automated blood pressure devices in cardiovascular disease prevention and management in primary health care settings. J Hum Hypertens. 2023;37(2):126-129.
- Sharman JE, Ordunez P, Brady T, Parati G, Stergiou G, Whelton PK, et al. The urgency to regulate validation of automated blood pressure measuring devices: a policy statement and call to action from the world hypertension league. J Hum Hypertens. 2023;37(2):155–9.
- Cheung AK, Whelton PK, Muntner P, Schutte AE, Moran AE, Williams B, et al. International Consensus on Standardized Clinic Blood Pressure Measurement - A Call to Action. Am J Med. 2023 May;136(5):438-445.e1.
- Picone DS, Campbell NRC, Schutte AE, Olsen MH, Ordunez P, Whelton PK, et al. Validation Status of Blood Pressure Measuring Devices Sold Globally. JAMA. 2022;327(7):680–1.
- Pan American Health Organization. HEARTS in the Americas Regulatory Pathway to the Exclusive Use of Validated Blood Pressure Measuring Devices. Washington D.C.; 2021. https://iris.paho.org/handle/10665.2/55382
- Lombardi C, Sharman JE, Padwal R, Picone D, Alcolea E, Ayala R, et al. Weak and fragmented regulatory frameworks on the accuracy of blood pressure-measuring devices pose a major impediment for the implementation of HEARTS in the Americas. J Clin Hypertens (Greenwich). 2020;22(12):2184-2191.
- Campbell NRC, Khalsa T, Ordunez P, Rodriguez Morales YA, Zhang XH, Parati G, et. al. Brief online certification course for measuring blood pressure with an automated blood pressure device. A free new resource to support World Hypertension Day Oct 17, 2020. J Clin Hypertens (Greenwich). 2020;22(10):1754-1756.
- Picone DS, Padwal R, Campbell NRC, Boutouyrie P, Brady TM, Olsen MH, et al. Accuracy in Measurement of Blood Pressure (AIM-BP) Collaborative. How to check whether a blood pressure monitor has been properly validated for accuracy. J Clin Hypertens (Greenwich). 2020;22(12):2167-2174.
- Sharman JE, O'Brien E, Alpert B, Schutte AE, Delles C, Hecht Olsen M, et al. Lancet Commission on Hypertension Group. Lancet Commission on Hypertension group position statement on the global improvement of accuracy standards for devices that measure blood pressure. J Hypertens. 2020;38(1):21-29.
- World Health Organization. WHO technical specifications for automated non-invasive blood pressure measuring devices with cuff. Geneva, Switzerland; 2020. https://www.who.int/publications/i/item/9789240002654

Access to treatment for hypertension and cardiovascular disease

- Schwalm JD, Joseph P, Leong D, Lopez-Lopez JP, Onuma O, Bhatt P, et al. Cardiovascular disease in the Americas: optimizing primary and secondary prevention of cardiovascular disease series: cardiovascular disease in the Americas. Lancet Reg Health Am. 2025;42:100964.
- Souza KM, Giron N, Vallini J, Hallar K, Ordunez P, Rosende A, et al. Barriers to access to antihypertensive medicines: insights from the HEARTS initiative in latin American and Caribbean region. J Pharm Policy Pract. 2024;17(1).
- Moran AE, Aimiosior O, Gupta R, Pathni A, Sahoo SK, Dessie G, Osi K, Zhang X, Banigbe B, Garg R, Frieden TR. Integrated Antihypertensive and Statin Treatment Protocols for Cardiovascular Disease Prevention in Low- and Middle-Income Countries. Glob Heart. 2024;19(1):93.
- Abdalla M, Bolen SD, Brettler J, Egan BM, Ferdinand KC, Ford CD, Lackland DT, Wall HK, Shimbo D; American Heart Association and American Medical Association. Implementation Strategies to Improve Blood Pressure Control in the United States: A Scientific Statement From the American Heart Association and American Medical Association. Hypertension. 2023;80(10):e143-e157.
- Cohn J, Bygrave H, Roberts T, Khan T, Ojji D, Ordunez P. Addressing Failures in Achieving Hypertension Control in Low- and Middle-Income Settings through Simplified Treatment Algorithms. Glob Heart. 2022 Apr 12;17(1):28. doi: 10.5334/gh.1082. PMID: 35586744; PMCID: PMC9009360.
- Al-Makki A, DiPette D, Whelton PK, Murad MH, Mustafa RA, Acharya S, et al. Hypertension Pharmacological Treatment

in Adults: A World Health Organization Guideline Executive Summary. Hypertension. 2022;79(1):293-301.

- Campbell NRC, Paccot Burnens M, Whelton PK, Angell SY, Jaffe MG, Cohn J, et al. 2021 World Health Organization guideline on pharmacological treatment of hypertension: Policy implications for the region of the Americas. Lancet Reg Health Am. 2022;9:None.
- Frieden TR, Moran AE, Garg R. HEARTS in the Americas: saving lives from the world's deadliest disease. Rev Panam Salud Publica. 2022;46:e171.
- Etienne CF. Scaling up cardiovascular disease management in primary care through HEARTS in the Americas. Rev Panam Salud Publica. 2022;46:e157
- Luciani S, Agurto I, Holder R, Caixeta R, Hennis AJ. Integrated approach for noncommunicable disease management in the Americas. Rev Panam Salud Publica. 2022 Sep 2;46:e154.
- Giron N, Lim C, Vallini J, Hallar K. Avanzando para mejorar el acceso a los medicamentos y tecnologías sanitarias para las enfermedades cardiovasculares [Moving toward improved access to medicines and health technologies for cardiovascular disease]. Rev Panam Salud Publica. 2022;46:e156.
- Flood D, Edwards EW, Giovannini D, Ridley E, Rosende A, Herman WH, Jaffe MG, DiPette DJ. HEARTS como herramienta para integrar el manejo de la hipertensión y la diabetes en los entornos de atención primaria de salud [Integrating hypertension and diabetes management in primary health care settings: HEARTS as a tool]. Rev Panam Salud Publica. 2022;46:e213.
- World Health Organization. Guideline for the pharmacological treatment of hypertension in adults. Geneva, Switzerland; 2021. https://www.who.int/publications/i/item/9789240033986.
- Jeemon P, Séverin T, Amodeo C, Balabanova D, Campbell NRC, Gaita D, et al. World Heart Federation Roadmap for Hypertension - A 2021 Update. Glob Heart. 2021;16(1):63.
- DiPette DJ, Goughnour K, Zuniga E, Skeete J, Ridley E, Angell S, et al. Standardized treatment to improve hypertension control in primary health care: The HEARTS in the Americas Initiative. J Clin Hypertens (Greenwich). 2020;22(12):2285-2295.
- Husain MJ, Datta BK, Kostova D, Joseph KT, Asma S, Richter P, et al. Access to cardiovascular disease and hypertension medicines in developing countries: An analysis of essential medicine lists, price, availability, and affordability. J Am Heart Assoc. 2020;9(9):e015302.
- DiPette DJ, Skeete J, Ridley E, Campbell NRC, Lopez-Jaramillo P, Kishore SP, et al. Fixed-dose combination pharmacologic therapy to improve hypertension control worldwide: Clinical perspective and policy implications. J Clin Hypertens (Greenwich). 2019;21(1):4-15.
- Herrett E, Gadd S, Jackson R, Bhaskaran K, Williamson E, van Staa T, et al. Eligibility and subsequent burden of cardiovascular disease of four strategies for blood pressure-lowering treatment: a retrospective cohort study. Lancet. 2019;394(10199):663–71.
- Skeete J, Connell K, Ordunez P, DiPette DJ. The American College of Cardiology/American Heart Association 2017 hypertension guideline: Implications for incorporation in Latin America, the Caribbean, and other resource-limited settings. J Clin Hypertens (Greenwich). 2018;20(9):1342-1349.

Country reports on HEARTS in the Americas implementation

- Acevedo LM, Kim TT, Hutchinson B, Toro L, Escobar MC, Basu S, et al. Economic evaluation of the HEARTS standardized hypertension treatment program with fixed-dose combination pills in Chile primary care clinics. Value Health. 2025:S1098-3015(25)02427-1.
- Durán M, Ugel E, Ojeda-Cáceres H, Bonelli A, Saglimbeni M, Rodríguez Araque E, et al. Evaluación de la implementación de la iniciativa HEARTS en una comunidad rural de Venezuela, 2023 [Evaluation of Implementation of the HEARTS Initiative in a Rural Community in Venezuela, 2023]. Rev Panam Salud Publica. 2024;48:e53.
- Valdés González Y, Morejón Giraldoni A, Pérez Perea L, Jiménez Chiquet A, López Lima C, Campbell N, Giraldo G. La Iniciativa HEARTS en Cuba: experiencias tras 5 años de implementación [The HEARTS Initiative in Cuba: experience of five years of implementation]. Rev Panam Salud Publica. 2022;46:e176.
- Michea L, Toro L, Alban N, Contreras D, Morgado P, Paccot M. et al. Eficacia de una estrategia estandarizada y simplificada para tratamiento de la hipertensión arterial en Chile: la Iniciativa HEARTS en las Américas [Efficacy of a standardized and simplified strategy for the treatment of high blood pressure in Chile: the HEARTS Initiative in the Americas]. Rev Panam Salud Publica. 2022 Sep 6;46:e138.
- Rodriguez G, Rosende A, Prado C, Cejas Mariño R, Irazola V, DiPette D, Orias M, Giraldo Arcila G, Laspiur S. Implementación de la Iniciativa HEARTS en Argentina: primeros resultados [Implementation of the HEARTS Initiative in Argentina: initial results]. Rev Panam Salud Publica. 2022;46:e181.
- Doon R, Malcolm T, Lewis Y, Holder L, Gulston L, Hamid A, Best A, Mitchell R, Lalla P. Improving cardiovascular health with the patient-centered, integrated primary care HEARTS model in Trinidad and Tobago. Rev Panam Salud Publica. 2022;46:e169.
- Casales-Hernández MG, Molina-Cuevas V, Gloria-Hernández LE, Díaz-Aguilera MA, Malo-Serrano HM. Resultados preliminares de la Iniciativa HEARTS en México: facilitadores y barreras de los sistemas de información [Preliminary results of the HEARTS Initiative in Mexico: facilitators and barriers in information systems]. Rev Panam Salud Publica. 2022;46:e167.
- Chivardi C, Hutchinson B, Molina V, Moreno E, Fajardo I, Giraldo-Arcila GP, Malo HM, Ordunez P, Rodríguez-Franco R, Moran AE, Kostova D. Assessing costs of a hypertension program in primary care: evidence from the HEARTS program in Mexico. Rev Panam Salud Publica. 2022;46:e144.
- Michea L, Toro L, Alban N, Contreras D, Morgado P, Paccot M, et al. Eficacia de una estrategia estandarizada y simplificada para tratamiento de la hipertensión arterial en Chile: la Iniciativa HEARTS en las Américas [Efficacy of a standardized and simplified strategy for the treatment of high blood pressure in Chile: the HEARTS Initiative in the Americas]. Rev Panam Salud Publica. 2022 Sep 6;46:e138.
- Silva P, Benavides C, Barake F, Neira C, Aguilera C, Hernández H, et al. Perfil de compra de dispositivos de medición de

presión arterial, Chile 2018-2020 [Profile of Blood pressure measurement devices purchased in Chile 2018-2020]. Rev Panam Salud Publica. 2022;46:e132.
- Campbell NR, Padwal R, Tsuyuki RT, Leung AA, Bell A, Kaczorowski J, Tobe SW. Altibajos del control de la hipertensión en Canadá: factores críticos y lecciones aprendidas. Rev Panam Salud Publica. 2022;46:e141.
- Barake F, Paccot M, Rivera M, Neira C, Reyes V, Escobar MC. Chile's public healthcare sector hypertension control rates before and during the pandemic and HEARTS implementation. Rev Panam Salud Publica. 2022;46:e126.
- Rivas Rivas S, Serna Tobón DC, Mahecha Gallego KY, Tejada Cardona MP, Castrillón Spitia JD, Moreno Gutierrez PA, Agudelo Ramírez A. Impacto de la Iniciativa HEARTS en una institución de salud de segundo nivel en Colombia [Impact of the HEARTS Initiative in a second-level health institution in Colombia]. Rev Panam Salud Publica. 2022;46:e152.
- González N, Vega L, Gloria L, Molina V, Malo M. Diseño de implementación del módulo de hábitos y estilos de vida saludables de HEARTS en el primer nivel de atención en México [Implementation design of the HEARTS healthy-lifestyle counselling module at the first level of care in Mexico]. Rev Panam Salud Publica. 2022;46:e200.
- Philbert SC, Lawrence-Williams P, Gebre Y, Hutchinson ML, Belmar-George S. Improving cardiovascular health in primary care in Saint Lucia through the HEARTS Initiative. Rev Panam Salud Publica. 2022;46:e128.
- Hancco Saavedra J, Pérez Jiménez V. La voluntad política y la gobernanza: claves en la implementación de HEARTS en el Perú [Political will and governance: keys to Implementing HEARTS in Peru]. Rev Panam Salud Publica. 2022;46:e85.
- Champagne BM, Antonio Ochoa E, Khanchandani HS, Schoj V. Civil society's role in improving hypertension control in Latin America. Rev Panam Salud Publica. 2022;46:e165.
- Valdés González Y, Campbell NRC, Pons Barrera E, Calderón Martínez M, Pérez Carrera A, Morales Rigau JM, et al. Implementation of a community-based hypertension control program in Matanzas, Cuba. J Clin Hypertens (Greenwich). 2020;22(2):142-149.

HEARTS implementation worldwide

- Goupil R, Tsuyuki RT, Santesso N, Terenzi KA, Habert J, Cheng G, et al. Hypertension Canada guideline for the diagnosis and treatment of hypertension in adults in primary care. CMAJ. 2025;197(20):E549-E564.
- Frieden TR, Garg R, Moran AE, Whelton PK. Improved hypertension care requires measurement and management in health facilities, not mass screening. Lancet. 2025;405(10492):1879-1882.
- Matsushita K, Angell SY, Appel LJ, Bygrave H, Cohn J, Kalyesubula R, et al. Priorities for Research on Hypertension Care Delivery: A WHO Report Executive Summary. Hypertension. 2025;82(6):971-976.
- Dessie GA, Beyene MH, Sibhatu MK, Bangibe B, Belbase B, Samarah D, Kebede HG, Mebrhatu AH, Getachew H, Alemayue EA, Worku A, Moran AE. Factors Associated with Hypertension Care Follow-Up in the Ethiopia HEARTS Program. Glob Heart. 2025;20(1):20.
- Abrar A, Hu X, Akhtar J, Jubayer S, Noor Nabi Sayem M, Sultana S, et al. Evaluation of the World Health Organization-HEARTS hypertension control package in Bangladesh: a quasi-experimental trial. Heart. 2024;110(17):1090-1098.
- Gupte, S.S., Sachdeva, A., Kabra, A. et al. Private provider practices and incentives for hypertension management in rural and peri-urban Telangana, India– a qualitative study. BMC Health Serv Res 24, 1206 (2024).
- Satheesh G, Dhurjati R, Huffman MD, Rosende A, Rodgers A, Prabhakaran D, et al. Standardized treatment protocols for hypertension: global availability, characteristics, and alignment with the hypertension guideline recommendations. J Hypertens. 2024;42(5):902-908.
- Wellmann IA, Ayala LF, Valley TM, et al. Evaluating the World Health Organization's Hearts Model for Hypertension and Diabetes Management: A Pilot Implementation Study in Guatemala. Global Heart. 2025; 20(1): 9.
- Moran AE, Gupta R; Global Hearts Initiative Collaborators. Implementation of Global Hearts Hypertension Control Programs in 32 Low- and Middle-Income Countries: JACC International. J Am Coll Cardiol. 2023 Nov 7;82(19):1868-1884.
- Sahoo SK, Pathni AK, Krishna A, Sharma B, Cazabon D, Moran AE, Hering D. Financial implications of protocol-based hypertension treatment: an insight into medication costs in public and private health sectors in India. J Hum Hypertens. 2023;37(9):828-834.
- Pickersgill SJ, Msemburi WT, Cobb L, Ide N, Moran AE, Su Y, Xu X, Watkins DA. Modeling global 80-80-80 blood pressure targets and cardiovascular outcomes. Nat Med. 2022;28(8):1693-1699.
- Moran AE, Farrell M, Cazabon D, Sahoo SK, Mugrditchian D, Pidugu A, et al. Building the health-economic case for scaling up the WHO-HEARTS hypertension control package in low- and middle-income countries. Rev Panam Salud Publica. 2022;46:e140.
- Ogungbe O, Cazabon D, Ajenikoko A, Jeemon P, Moran AE, Commodore-Mensah Y. Determining the frequency and level of task-sharing for hypertension management in LMICs: A systematic review and meta-analysis. EClinicalMedicine. 2022;47:101388.
- Marklund M, Cherukupalli R, Pathak P, Neupane D, Krishna A, Wu JHY, et al. Hypertension treatment capacity in India by increased workforce, greater task-sharing, and extended prescription period: a modelling study. Lancet Reg Health Southeast Asia. 2022;10:100124.
- Ojji DB, Baldridge AS, Orji IA, Shedul GL, Ojo TM, Ye J, et al. Characteristics, treatment, and control of hypertension in public primary healthcare centers in Nigeria: baseline results from the Hypertension Treatment in Nigeria Program. J Hypertens. 2022;40(5):888-896.
- Ogungbe O, Cazabon D, Ajenikoko A, Jeemon P, Moran AE, Commodore-Mensah Y. Determining the frequency and level of task-sharing for hypertension management in LMICs: A systematic review and meta-analysis. EClinicalMedicine. 2022;47:101388.
- Cazabon D, Farrell M, Gupta R, Joseph L, Pathni AK, Sahoo S, Kunwar A, Elliott K, Cohn J, Frieden TR, Moran AE. A simple six-step guide to National-Scale Hypertension Control Program implementation. J Hum Hypertens. 2022 Jul;36(7):591-603.

- Olsen MH, Neupane D, Cobb LK, Frieden TR, Hall B, Lackland DT, et al. Global cardiovascular disease prevention and management: A collaboration of key organizations, groups, and investigators in low- and middle-income countries. J Clin Hypertens (Greenwich). 2020;22(8):1293-1295.
- Frieden TR, Varghese CV, Kishore SP, Campbell NRC, Moran AE, Padwal R, Jaffe MG. Scaling up effective treatment of hypertension-A pathfinder for universal health coverage. J Clin Hypertens (Greenwich). 2019;21(10):1442-1449.

Burden of hypertension and cardiovascular diseases

- Martinez R, Muñoz-Venturelli P, Ordunez P, Fregni F, Abanto C, Alet M, et al. Risk and impact of stroke across 38 countries and territories of the Americas from 1990 to 2021: a population-based trends analysis from the Global Burden of Disease Study 2021. Lancet Reg Health Am. 2025;43:101017.
- Joseph P, Lanas F, Roth G, Lopez-Jaramillo P, Lonn E, Miller V, et al. Cardiovascular disease in the Americas: the epidemiology of cardiovascular disease and its risk factors. Lancet Reg Health Am. 2025;42:100960.
- World Health Organization. Global Report on hypertension. The race against a silent killer. 2023. Geneva, Switzerland; 2023. https://www.who.int/publications/i/item/9789240081062
- World Heart Report 2023: Confronting the World's Number One Killer. Geneva, Switzerland. World Heart Federation. 2023. https://world-heart-federation.org/wp-content/uploads/World-Heart-Report-2023.pdf
- Martinez R, Soliz P, Campbell NRC, Lackland DT, Whelton PK, Ordunez P. Association between population hypertension control and ischemic heart disease and stroke mortality in 36 countries of the Americas, 1990-2019: an ecological study. Rev Panam Salud Publica. Spec Issue Hear. 2022;46.
- Sosa Liprandi Á, Baranchuk A, López-Santi R, Wyss F, Piskorz D, Puente A, Ponte-Negretti CI, Muñera-Echeverri A, Piñeiro DJ. El control de la hipertensión arterial, una asignatura pendiente [Control of arterial hypertension: a pending issue]. Rev Panam Salud Publica. 2022 Sep 16;46:e147.
- Martinez R, Morsch P, Soliz P, Hommes C, Ordunez P, Vega E. Life expectancy, healthy life expectancy, and burden of disease in older people in the Americas, 1990-2019: a population-based study. Rev Panam Salud Publica. 2021;45:e114.
- NCD Risk Factor Collaboration (NCD-RISC). Worldwide trends in hypertension prevalence and progress in treatment and control from 1990 to 2019: a pooled analysis of 1201 population-representative studies with 104 million participants. Lancet. 2021;398(10304):957-980.
- Zhou B, Perel P, Mensah GA, Ezzati M. Global epidemiology, health burden and effective interventions for elevated blood pressure and hypertension. Nat Rev Cardiol. 2021;18(11):785–802.
- Schutte AE, Srinivasapura Venkateshmurthy N, Mohan S, Prabhakaran D. Hypertension in Low- and Middle-Income Countries. Circ Res. 2021;128(7):808–26.
- Zhou B, Carrillo-Larco RM, Danaei G, Riley LM, Paciorek CJ, Stevens GA, et al. Worldwide trends in hypertension prevalence and progress in treatment and control from 1990 to 2019: a pooled analysis of 1201 population-representative studies with 104 million participants. Lancet. 2021;398(10304):957–80.
- Martinez R, Lloyd-Sherlock P, Soliz P, Ebrahim S, Vega E, Ordunez P, et al. Trends in premature avertable mortality from non-communicable diseases for 195 countries and territories, 1990–2017: a population-based study. Lancet Glob Heal. 2020;8(4):e511–23.
- Martinez R, Soliz P, Mujica OJ, Reveiz L, Campbell NRC, Ordunez P. The slowdown in the reduction rate of premature mortality from cardiovascular diseases puts the Americas at risk of achieving SDG 3.4: A population trend analysis of 37 countries from 1990 to 2017. J Clin Hypertens. 2020;22(8):1296–309.
- Martinez R, Lloyd-Sherlock P, Soliz P, Ebrahim S, Vega E, Ordunez P, et al. Trends in premature avertable mortality from non-communicable diseases for 195 countries and territories, 1990–2017: a population-based study. Lancet Glob Heal. 2020;8(4):e511–23.
- Ebrahim S, Ordunez P, Lloyd-Sherlock P, McKee M, Martinez R, Soliz P. Improving the indicator for premature deaths from noncommunicable diseases. Bull World Health Organ. 2020;98(6):438-440.
- Mills KT, Stefanescu A, He J. The global epidemiology of hypertension. Nat Rev Nephrol. 2020;16(4):223-237.
- Lloyd-Sherlock P, Ebrahim S, Martinez R, McKee M, Ordunez P. Reducing the cardiovascular disease burden for people of all ages in the Americas region: analysis of mortality data, 2000-15. Lancet Glob Health. 2019;7(5):e604-e612.
- Geldsetzer P, Manne-Goehler J, Marcus ME, Ebert C, Zhumadilov Z, Wesseh CS, et al. The state of hypertension care in 44 low-income and middle-income countries: a cross-sectional study of nationally representative individual-level data from 1·1 million adults. Lancet. 2019;394(10199):652–62.
- Lopez AD, Adair T. Is the long-term decline in cardiovascular-disease mortality in high-income countries over? Evidence from national vital statistics. Int J Epidemiol. 2019;48(6):1815–23.
- Campbell NRC, Gonzalez YV, Ordunez P. The burden of hypertension in Cuba. Lancet Public Health. 2019 Feb;4(2):e79-e80. Erratum in: Lancet Public Health. 2019;4(2):e88.
- Martinez R, Soliz P, Caixeta R, Ordunez P. Reflection on modern methods: years of life lost due to premature mortality-a versatile and comprehensive measure for monitoring non-communicable disease mortality. Int J Epidemiol. 2019;48(4):1367-1376.
- Lamelas P, Diaz R, Orlandini A, Avezum A, Oliveira G, Mattos A, et al. Prevalence, awareness, treatment and control of hypertension in rural and urban communities in Latin American countries. J Hypertens. 2019 Sep 1;37(9):1813–21.
- Olsen MH, Angell SY, Asma S, Boutouyrie P, Burger D, Chirinos JA, et al. A call to action and a lifecourse strategy to address the global burden of raised blood pressure on current and future generations: the Lancet Commission on hypertension. Lancet. 2016;388(10060):2665-2712.

Supplementary Box 2. HEARTS virtual courses

- **Hypertension control drivers at primary health care centers**
  https://campus.paho.org/en/course/HypertensionCntrlDrivers

- **Virtual Course on accurate automated blood pressure measurement**
  https://campus.paho.org/en/course/Blood-Pressure-Measurement

- **[Apoyo a los equipos de atención primaria de la salud para el uso efectivo de medicamentos antihipertensivos]**
  https://campus.paho.org/es/curso/Medicamentos-Antihipertensivos

- **Home blood pressure monitoring. Promoting patient self-measurement**
  https://campus.paho.org/en/course/home-blood-pressure-monitoring

- **[HEARTS en las Américas: Mejora continua de la calidad para los equipos de atención primaria]**
  https://campus.paho.org/es/curso/hearts-mejora-continua-calidad_equipos-APS
