# Supplementary material for: HEARTS quality: a policy framework to strengthen hypertension and cardiovascular risk management in primary healthcare—insights from HEARTS in the Americas
Source: Lancet Reg Health Am. 2025 Dec 1;53:101311. doi: 10.1016/j.lana.2025.101311 (PMC12719693; doi:10.1016/j.lana.2025.101311)
Supplement: Supplementary Material 2 [file mmc2.docx]

**HEARTS Quality: A policy framework to strengthen hypertension and cardiovascular risk management in primary health care—Insights from HEARTS in the Americas.**

**Supplementary Material**

**Table of content**

Supplementary Summary in Spanish………………………………………………………………………..………… 2

**Editorial disclaimer**

The translation of the Summary was submitted by the authors, and we reproduce it as supplied. It has not been peer reviewed. Our editorial processes have only been applied to the original version in English, which should serve as a reference for this manuscript.

Supplementary Summary in Spanish

Resumen

HEARTS en las Américas constituye la implementación de mayor escala de la iniciativa ‘HEARTS Global’ de la OMS, alcanzando la participación de 33 países, 28 de los cuales han adoptado vías clínicas estandarizadas, y cerca de 10.000 establecimientos de atención primaria de salud. A pesar de los avances, la atención fragmentada, la disponibilidad limitada de dispositivos validados para la medición de la presión arterial, el acceso restringido a medicamentos esenciales y la debilidad de los sistemas de garantía de la calidad, continúan obstaculizando el control de la hipertensión y la gestión del riesgo cardiovascular. En respuesta, la OPS y los países participantes desarrollaron el Marco de Calidad de HEARTS. Partiendo de las experiencias de implementación a lo largo de la Región, este modelo sintetiza la evidencia global y las lecciones aprendidas en América Latina y el Caribe. Co-diseñado por ministerios de salud, prestadores de servicios y expertos internacionales, este marco de calidad traduce las estrategias de HEARTS en objetivos operativos ejecutables a nivel del sistema de salud. Los indicadores de resultado y las metas de implementación, claramente definidos, fomentan la institucionalización, la mejora continua de la calidad y el fortalecimiento de la atención primaria de salud, apoyando la expansión de HEARTS y la obtención de resultados equitativos. Con la contextualización adecuada, el Marco de Calidad de HEARTS ofrece una hoja de ruta práctica para que los países fuera de la Región avancen hacia una atención de las enfermedades crónicas basada en la Atención Primaria de Salud.
